# Sex differences in porcine left ventricular myocardial remodeling due to right ventricular pacing

**DOI:** 10.1186/s13293-015-0048-4

**Published:** 2015-12-10

**Authors:** Liliana Kiczak, Alicja Tomaszek, Urszula Pasławska, Jacek Bania, Agnieszka Noszczyk-Nowak, Piotr Skrzypczak, Robert Pasławski, Maciej Zacharski, Adrian Janiszewski, Piotr Kuropka, Piotr Ponikowski, Ewa A. Jankowska

**Affiliations:** Research and Development Centre, Regional Specialist Hospital in Wroclaw, Kamienskiego Street 73a, 51-124 Wroclaw, Poland; Department of Biochemistry, Pharmacology and Toxicology, Faculty of Veterinary Medicine, Wroclaw University of Environmental and Life Sciences, Norwida Street 31, 50-375 Wroclaw, Poland; Department of Heart Diseases, Wroclaw Medical University, Weigla Street 5, 50-981 Wroclaw, Poland; Department of Internal Diseases and Clinic of Diseases of Horses, Dogs and Cats, Faculty of Veterinary Medicine, Wroclaw University of Environmental and Life Sciences, Grunwaldzki Sq. 47, 50-366 Wroclaw, Poland; Department of Food Hygiene and Consumer Health Protection, Faculty of Veterinary Medicine, Wroclaw University of Environmental and Life Sciences, Norwida Street 31, 50-375 Wroclaw, Poland; Department of Surgery, Faculty of Veterinary Medicine, Wroclaw University of Environmental and Life Sciences, Grunwaldzki Sq. 51, 50-366 Wroclaw, Poland; Department and Clinic of Internal and Occupational Diseases, Hypertension and Clinical Oncology, Wroclaw Medical University, Borowska Street 213, 50-556 Wroclaw, Poland; Department of Animal Physiology and Biostructure, Faculty of Veterinary Medicine, Wroclaw University of Environmental and Life Sciences, Wroclaw, Poland

**Keywords:** Experimental model of heart failure, Myocardial remodeling, Sex, Extracellular matrix turnover

## Abstract

**Background:**

Although sex differences in heart failure (HF) prevalence and severity have been recognized, its molecular mechanisms are poorly understood. We used a tachycardia-induced cardiomyopathy model to determine the sex specific remodeling pattern in male and female adult pigs.

**Methods:**

We compared the echocardiographic and molecular measures of myocardial remodeling in 19 male and 12 female pigs with chronic symptomatic systolic HF due to right ventricle (RV) pacing (170 bpm) and 6 male and 5 female sham-operated controls. Males achieved subsequent HF stages earlier than females.

**Results:**

The progression of symptomatic HF was associated with the reduction of the left ventricle (LV) ejection fraction in both sexes (all *p* < 0.05). A significant LV dilatation occurred only in males (*p* < 0.001). The HF development was accompanied by an increased pro-hypertrophic factor GATA4 and TGF-β1 messenger RNA (mRNA) expression in the LV only in male pigs (all *p* < 0.01). The total gelatinolytic activity in LV was higher in males than females (irrespective of HF, *p* < 0.05), and the HF progression was associated with a reduced total gelatinolytic activity (*p* < 0.05) in the LV only in males. No differences in LV myocardial collagen content were found between HF groups and sexes. Cardiomyocyte cross-sectional diameter was significantly smaller in male hearts as compared to female (*p* < 0.05).

**Conclusions:**

Male and female porcine hearts respond differently to RV pacing. Males, most likely due to a higher extracellular matrix turnover, demonstrated a significant LV dilatation, followed by a strong induction of pro-hypertrophic program, and an earlier development of symptomatic HF.

**Electronic supplementary material:**

The online version of this article (doi:10.1186/s13293-015-0048-4) contains supplementary material, which is available to authorized users.

## Background

There are prominent sex differences in the prevalence and severity of heart failure (HF) reported in human clinical studies [1]. Gender-related disparities in the outcome of HF as well as its clinical presentation are observed [2–5]. Women more commonly have HF with a preserved left ventricle ejection fraction, while the systolic function more frequently deteriorates in men [1]. The causes of these differences remain unclear. Observational clinical and post-mortem studies suggest the presence of important differences in cardiac remodeling between men and women [6]. Rodent experimental models of pressure overload [7], volume overload [8], and myocardial infarction (MI) [9] also confirmed sex differences in myocardial remodeling in response to an injury. Gene expression profiling in a rodent HF model [10] as well as in end-stage failing human hearts [3] has revealed greater upregulation of matrix-related genes in males than in females.

Although rodents remain the species of choice for current experimental studies [11], there are significant differences in the anatomy and physiology of their cardiovascular system in comparison to large mammals and human [12]. What is more, there are significant differences in the cardiac function between different mouse strains [13]. To our knowledge, there are no available data concerning sex differences in myocardial remodeling in the course of HF in large animal models. Furthermore, rodent experimental models of HF examining gender differences lasted up to 12 weeks, during which was collected data only at end-points, without showing the subsequent stages leading to the overt HF [7–9]. Owing to the fact that differential gene expression profiles of men and women with new-onset HF [14] seem to disappear during later stages of the disease [15], it is important to study the sex differences in the initial stages of HF.

Porcine hearts, exhibiting a gross anatomic structure very similar to that of humans, seem to be suitable for translational studies [12]. Right ventricle (RV) pacing-induced tachycardia applied in pigs was shown to be an effective trigger for the development of progressive left ventricle (LV) dilatation and dysfunction as well as the activation of neurohormonal systems [16, 17]. We have developed an experimental model of symptomatic chronic HF in female pigs based on relatively slow, long-term RV pacing. In this model, we have been able to induce myocardial dysfunction confirmed both by echocardiography and molecular methods, accompanied by an occurrence of clinical features of the HF syndrome [18].

Our goal was to use a tachycardia-induced cardiomyopathy (TIC) model to determine a sex-specific remodeling pattern in age-matched male and female adult pigs. On the basis of a comprehensive clinical evaluation, each animal was assigned to mild, moderate, or severe HF groups, and presented for euthanasia at each stage of HF. This study design accomplishes both longitudinal and end-point data analysis.

## Methods

### Studied animals and the protocol of the experiment

The study was performed on 42 Polish Large White breed adult pigs of 8-month-old siblings, 27 males (100.1 ± 21.2 kg) and 17 females (87.6 ± 11.7 kg). Initial heart rates for males were 79.12 ± 12.28, for females 82.17 ± 17, the difference was not statistically significant.

All animals received appropriate care in compliance with the *Guide for the Care and Use of Laboratory Animals* as published by the National Institutes of Health (NIH publication No. 85-23, revised in 1996). All experiments were performed in compliance with the Bioethical Committee of the Wroclaw University of Environmental and Life Sciences guidelines for the experimentation on animals.

All procedures and echocardiography measurements were performed during anesthesia administered according to the protocol described below, with food restriction for 12 h and water restriction for 4 h prior to the procedure. Pigs were anesthetized using a modified protocol described by Goldmann et al. [19]. In brief, animals were premedicated with an intramuscular injection of 1 mg/m^2^ body surface area (BSA) medetomidine hydrochloride (Cepetor, CP-Pharma, Germany), 5 mg/m^2^ BSA of midazolam (Midanium, WZF Polfa, Warsaw, Poland), and 264 mg/m^2^ BSA of ketamine (Bioketan, Vetoquinol Biowet, Poland) in a mixing syringe. An ear vein was punctured for the placement of a catheter for an intravenous induction of propofol (Propofol 1 % MCT/LCT Fresenius, Fresenius Kabi, Germany) at 2–5 mg/kg body weight (BW). Following intubation (8.5 Charriere tubes with blunt-tipped plastic guide wire) [20], anesthesia was maintained by continuous infusions of 1–3 μg/h per kilogram BW fentanyl (Fentanyl WZF, WZF Polfa, Warsaw, Poland) and inhalation of isoflurane (1.5–2 % vol) (Aerrane, Baxter, Warsaw, Poland). Monitoring of the basal life functions (ECG, end-tidal CO_2_, oxygen saturation, noninvasive blood pressure) was carried out using LIFEPAK 12 Defibrillator/Monitor (Medtronic, Warsaw, Poland). A single-chamber pacemaker (SENSIA SESR01, Medtronic) was implanted in each of the 42 pigs under control of a fluoroscope (Ziehm 8000, Ziehm Imaging, Nuernberg, Germany). A bipolar screw-in pacing transvenous lead (CAPSUREFIX NOVUS 58 cm, Medtronic) was inserted into the left internal jugular vein and positioned in the myocardium at the right ventricular apex. The lead was attached to the pacemaker, and the pacing system was placed in a subcutaneous pocket. Each pig was administered an antibiotic intramuscularly for infection prophylaxis for 10 days. The animals were allowed a 2-week recovery period, and the pacemakers were programmed for sequential right ventricular pacing at 170 bpm (beat per minute) in randomly chosen animals (19 males, 12 females). Sham-operated animals served as controls (6 males, 5 females).

### Performed assessment schedule

All animals remained under everyday clinical care. There was no difference in the measurement protocol between paced and non-paced pigs. The assessment was regularly performed at the end of every month and comprised (1) clinical assessments with an evaluation of HF signs and symptoms (for details see below) and (2) transthoracic echocardiography (for details see below).

The following aspects of the animal’s physical condition were evaluated monthly on a 0–3 scale: appetite (0—normal behavior; 1—decreased appetite; 2—significantly decreased appetite; 3—no appetite), interest in surroundings (0—normal interest in surroundings; 1—decreased interest in surroundings; 2—significantly decreased interest in surroundings; 3—no interest in surroundings), physical activity willingness (after forcing) (0—normal behavior; 1—decreased willingness to be physically active; 2—significantly decreased willingness to be physically active; 3—no willingness to be physically active). The monthly clinical assessment of heart insufficiency comprised of the following features at rest: dyspnea, ascites, snout, and ears cyanosis. The following were observed after exertion: the presence of a shortening of breath, dyspnea, redness of the snouts and ears, lying down. Each feature was evaluated on 0–3 scale (0—no clinical signs of heart insufficiency at rest and after exertion; 1—mild, 2—moderate; and 3—severe increase of heart insufficiency signs at rest and after exertion). All points for each pig were summarized, and the arithmetic average was calculated. The following scoring for HF categorization was used: mild HF (0–1), moderate HF (1.1–2), and severe HF (2.1–3). The study was designed prospectively in such a way that animals developing the consecutive stages of HF (mild, moderate, and severe) during the experiment were presented for euthanasia. Control animals underwent euthanasia parallel to TIC pigs and were randomly selected for this procedure. The pigs were euthanized with an overdose of pentobarbital. Tissue sections from the LV free wall were taken and immediately frozen in liquid nitrogen. At the same time, separate sections for standard histology were immersed in a 4 % paraformaldehyde solution and stored for the further assessments.

### Transthoracic echocardiography

Before transthoracic echocardiography (ECG) measurements, each animal was anesthetized and the pacemaker was deactivated for approximately 30 min. Transthoracic echocardiography with simultaneous ECG recordings was performed using an imaging ultrasound system (Aloka 4000+ with a 3.5-MHz phased-array transducer, Aloka Company, Tokio, Japan). Right parasternal views were readily visible in all animals, whereas the left apical view was not available. Two-dimension and direct M-mode echocardiography was performed in the right parasternal area in a left lateral decubitus position. Diastolic measurements were taken at the onset of the QRS complex of the ECG. Systolic measurements were taken at the end of the T wave. The ratio of the left atrium to the diameter of the aorta (LA/Ao) was measured in diastole from the 2-D short axis at the level of the aortic valve. The LV end diastolic diameter (LVEDD, cm) was measured using the leading-edge method based on at least three consecutive cardiac cycles as recommended by the American Society for Echocardiography [21]. The end-diastolic and end-systolic thickness of the left ventricle posterior wall (LVPWd and LVPWs) was measured from images that were collected from a right long-axis four-chamber view. With the use of the [22], LV end-diastolic (LVEDV, ml) and the end-systolic volume (LVESV, ml) as well as the LV ejection fraction (LVEF, %) and stroke volume (SV, ml) were computed. Relative wall thickness at end diastole (RWTd) was calculated using the equation 2 × LVPWd/LVEDD.

Left ventricular diastolic functions were measured by pulsed-waved tissue Doppler ultrasonography. For this purpose, the Doppler gate was placed over the basal segment of the left ventricular free wall just below the mitral annulus in the parasternal short-axis view. Measurements of myocardial velocities during early diastole or early filling (Em, m/s) and late diastole or atrial contraction (Am, m/s) were made, and the Em/Am ratio was calculated in all pigs. Tracings were recorded at a sweep speed 100 mm/s, and measurements were averaged for three separate heart beats.

### Quantitative RT-PCR

Total RNA was prepared from 30-mg samples of porcine LV tissue using the RNeasy Fibrous Tissue Mini Kit (Qiagen, Wroclaw, Poland) according to the manufacturer’s instructions. The protocol included on-column DNAse digestion to remove the genomic DNA. First-strand cDNA was synthesized using a SuperScript III First-Strand Synthesis System with an oligo(dT)_20_ primer (Invitrogen, Warsaw, Poland).

Based on the genomic and complementary DNA (cDNA) sequences, the primers for BNP (B-type natriuretic peptide), GATA4 (GATA binding protein 4) TGF-β1 (tissue growth factor β1), MMP9 (matrix metalloproteinase 9), TIMP1 (tissue inhibitor of metalloproteinases type 1), NGAL (neutrophil gelatinase-associated lipocalin), Col1A1 (collagen, type I, alpha 1), Col1A2 (collagen type I, alpha 2), and Col3A1 (collagen type III, alpha 1) were designed with Molecular Beacon Software (Bio-Rad, Warsaw, Poland) (Table 1). The primers spanned exon junctions to prevent the amplification of genomic DNA. The glyceraldehyde-3-phosphate dehydrogenase (GAPDH) gene was chosen as a reference to normalize the differences in the amount of RNA and in the efficiency of reverse transcription (Table 1).

**Table 1:** Oligonucleotide primers used in RT-PCR experiments

Gene	Sequence 5′-3′	GenBank accession no.
GAPDH	TCACTGCCACCCAGAAGA TACCAGGAAATGAGCTTGAC	ABO38240
BNP	GATACAGGAGCTGCTGGAC GAGGACTTGGAAGATGCTACTGC	M23596
GATA4	CAGCAGCAGCGAAGAGATG CGAGAGGACCGGGTGGATGG	AY115491
TGF-β1	CTACTACGCCAAGGAGGTCAC GCCCGAGAGAGCAATACAGG	X12373
MMP9	CCACAGGCCCTCCTTCAG TGAACAGCAGCACCTTACC	NM001038004
NGAL	TTAAGAAATACTCTGGATTGC TACTCTTGGTTGTTGGAAAC	AK240091
TIMP1	AGCCAGGAGTTTCTCATAGC TCACAGCCAGCAGCATAG	NM213857
Col1A1	AGCGGAGAATACTGGATTGAC CGTGCCTCTTGTCCTTGG	AF201723
Col1A2	ATGCCGTGACTTGAGACTC CCTTGGTGGTAACTCCTTCC	AB237775
Col3A1	CGGACAAATAGAAAGCCTCATTAG GAAGTTCAGGATTGCCATAGC	DT322297

*GAPDH* glyceraldehyde-3-phosphate dehydrogenase, *BNP* B-type natriuretic peptide, *GATA4* GATA binding protein type 4, *TGF-β1* transforming growth factor β1, *MMP9* matrix metalloproteinase type 9, *NGAL* neutrophil gelatinase-associated lipocalin, *TIMP1* tissue inhibitor of metalloproteinases type 1, *Col1A1* collagen type 1 α1, *Col1A2* collagen type I α2, *Col3A1* collagen type III α1

The relative amounts of porcine GATA4, TGF-β1_,_ TIMP1, NGAL, Col1A1, Col1A2, and Col3A1 in LV samples were determined by quantitative real-time PCR using the iQ5 Optical System (Bio-Rad) with the SSoFast Eva Green Supermix (Bio-Rad). The reactions were performed under the following conditions: initial denaturation at 94 °C for 10 min, 35 cycles at 94 °C for 30 s, 58 °C for 30 s, followed by 72 °C for 1 min.

The relative amounts of porcine BNP, MMP9, and NGAL in the LV myocardium were determined using the quantitative real-time PCR using the iQ5 Optical System (Bio-Rad) with the Kapa Mix (KapaBiosystems, USA) as appropriate. The reactions were performed under the following conditions: initial denaturation at 94 °C for 10 min, 35 cycles at 94 °C for 30 s, 60 °C (BNP, NGAL) or 65 °C (MMP9) for 30 s, followed by 72 °C for 1 min.

All samples were performed in triplicates. The specificity of the PCR was determined by melt-curve analysis for each reaction. PCR products for each investigated gene were sequenced (Genomed, Warsaw, Poland) to confirm their identity. The amplification efficiency was estimated by running serial dilutions of a template. Successive dilutions were plotted against the appropriate *C*_t_ values to generate a standard curve. The slope calculated from the standard curve was used to determine the amplification efficiency (*E*) according to the formula: *E* = 10^−1/slope^. Since the amplification efficiencies for the target amplicons and GAPDH were not comparable, the Pfaffl method was used to determine the relative expression [23]. Messenger RNA (mRNA) expression was presented in arbitrary units (AU), where the sample from LV myocardium from one of the control pigs was chosen as the calibrator, and its mRNA expression was considered as 1.

### Assessment of myocardial collagen content

The extraction of collagens from tissue, due to its ability to form cross-linked fibrils, is very difficult and requires special conditions, i.e., acetic acid, high temperature, or pepsin digestion [24]. However, degraded, small fragments of collagen (after cleavage by matrix metalloproteinases, MMPs) are soluble in water [25] and can be extracted from the plasma [26] or tissues [27].

Water soluble collagen (containing presumably collagen fragments after degradation by MMPs) fraction in myocardium was assessed by modified, previously described method [28] using Sircol collagen assay (Biocolor, Poland). The Sirius red stain used in this assay specifically binds to the [Gly-X-Y] structure found in all collagen fibers [29]. LV tissue samples (approximately 30 mg) were homogenized in distilled water and centrifuged. Equal amounts of supernatants (10 μg of total protein) were added to triplicate wells of a 96-well microtiter plate. The samples were stained for 30 min with 100 μl Sircol dye reagent, then centrifuged for 30 min (2250×*g*, room temperature), and the plates were dried. The dye was solubilized in 100 μl of 0.1 M NaOH, and the plates were read by spectrophotometry at an absorbance of 540 nm. Acid soluble type 1 collagen in 0.5 M acetic acid was used as a positive control and to generate a standard curve. The amount of collagen per 10 μg total protein was obtained from the standard curve and multiplied by the total protein to give total water soluble collagen levels. Total water soluble collagen levels were divided by the initial LV wet weight to obtain the microgram of water soluble collagen per milligram of LV wet weight.

The total recoverable collagen content in myocardium was assessed using a modified, previously described method [28, 30]. LV tissue homogenates (approximately 30 mg) were digested for 24 h by porcine pepsin (200 μg/ml, Sigma-Aldrich, Poland) in 0.5 M acetic acid at 37 °C [30]. After, centrifugation 100 μl of the supernatant was removed and the collagen content was determined by using the Sircol collagen assay (Biocolor, Poland). Equal amounts of supernatants (10 μg of total protein) were added to triplicate wells of a 96-well microtiter plate. The samples were stained for 30 min with 100 μl Sircol Dye Reagent, then centrifuged for 30 min (2250×*g*, room temperature) after which the plates were dried. The dye was solubilized in 100 μl of 0.1 M NaOH, and the plates were read by spectrophotometry at an absorbance of 540 nm. Acid soluble type 1 collagen in 0.5 M acetic acid was used as a positive control and to generate a standard curve. The amount of total recoverable collagen per 10 μg total protein was obtained from the standard curve and was multiplied by the total protein to give total collagen levels. The total recoverable collagen levels were divided by the initial LV wet weight to obtain micrograms of total recoverable collagen per milligram of LV wet weight.

## Histological assessment of myocardial collagen content and myocyte diameter

### Histological analysis

The LV specimens were embedded in paraffin, and transverse and cross-sections 5-μm thick were stained with van Gieson (Elastica van Gieson Kit, Merck, Poland) to evaluate interstitial fibrosis. The van Gieson method gives the most selective staining of collagen fibers (Dhein 2005). The percent area of extracellular staining was computed from 15 random fields within the myocardium in order to exclude large epicardial arteries and veins and any cutting or compression artifact, and results were then averaged. Images were viewed on a Nikkon Eclipse 80i microscope. Digitally acquired images were analyzed with Nis-Elements AR analysis software (Nikon Instruments Inc. Poland).

To quantify myocyte diameter (at ×20 magnification fields), a point-to-point perpendicular line drawn across the cross-sectional area of the myocytes at the level of the nucleus and the diameter length was measured with the computer imaging software. A total of 50 myocytes/slide were measured from each tissue specimen, and the mean SD per section was noted.

### Total gelatinolytic activity

LV samples (100 mg) were homogenized in 200 μl of an ice-cold extraction buffer (50 mM Tris-HCl, 200 mM NaCl, 10 mM CaCl_2_, 1 % Triton X-100, pH 7.6) [31]. After incubation on ice (30 min) and a centrifugation at 9700×*g*, the supernatants were collected and stored on ice. Next, insoluble material was extracted twice during a 10-min incubation with 50 μl of an ice-cold extraction buffer, and supernatants from all extractions were combined. Protein quantification was performed using the Bradford reagent (Sigma-Aldrich), according to the manufacturer’s instructions.

Total gelatinolytic activity was determined using biotinylated gelatin as the substrate. Gelatin (Sigma-Aldrich) was biotinylated using (+)-biotin N-hydroxysuccinimide ester (Sigma-Aldrich) according to the manufacturer’s instructions. Gelatin-biotin (diluted in 50 mM Tris-HCl, 5 mM CaCl_2_, pH 7.5) was loaded onto a 96-well plate (1 μg/well, Maxisorp, Nunc, Poland) and incubated for 2 h at 37 °C. The plate was washed extensively with phosphate-buffered saline (PBS) containing 0.05 % (*v*/*v*) Brij (Sigma-Aldrich), and LV homogenates (5 μg) were loaded into the wells and incubated for 24 h at 37 °C. The plate was washed extensively with PBS containing 0.05 % (*v*/*v*) Brij, incubated with streptavidin-HRP (Sigma-Aldrich) for 10 min at room temperature, again washed and developed using the TMB substrate (3,3′,5,5′-tetramethylbenzidine, Sigma-Aldrich). After stopping the reaction, the plate was read at 450 nm. The *A*_450_ value for each animal was divided by the mean *A*_450_ value for sham-operated male pigs and multiplied by 100. Each sample was measured in triplicate.

To confirm the concentration dependency as well as specificity of the gelatin-biotin assay system, serial dilutions of a culture medium from the DH82 macrophage-like cell line [32] were used.

### Statistical analyses

The data were expressed as the mean and the standard error of the mean (SEM), unless otherwise indicated. All molecular and echocardiographic assessments were performed in triplicates. Indices of LV function at different time points (separately for control and TIC animals) were tested using one-way ANOVA. A two-way ANOVA was used for the identification of sex-dependent parameters such as expressed genes as well as echocardiography and extracellular matrix (ECM) turnover parameters in the comparison of four conditions (sham-operated female, sham-operated male, TIC female, TIC male). The two-way ANOVA tested also the interaction between HF (sham-operated/TIC) and sex (female/male). Relationships between echocardiographic as well as remodeling parameters and HF were analyzed using Spearman’s rank correlation coefficients (independently in males and females). Spearman’s rank correlation coefficients were used for all correlatory analyses. The statistical differences in mean values of echocardiography data, expressed genes, and ECM turnover between controls and subsequent HF groups were tested using the Mann-Whitney *U* test. A Student’s *t* test was used to asses the sex difference in analyzed data within the same animal group. Statistical analysis was performed using commercially available software (Statistica for Windows, version 9.1, StatSoft, Poland). Values of *p* < 0.05 were considered to be significant.

## Results

### Time course of the development of symptomatic heart failure in male and female pigs due to RV pacing

Thirty one pigs were randomly assigned for RV pacing, and 11 pigs were sham-operated. All animals were raised in the same conditions to reduce the influence of environmental factors on the results. All pigs that were included in the project completed the whole study protocol.

Nineteen male and 12 female pigs were RV-paced. Males developed symptoms of mild, moderate, and severe HF after 6 ± 2 (*n* = 7), 11 ± 3 (*n* = 7), and 18 ± 4 weeks (*n* = 5) of RV pacing, respectively (*p* < 0.001), and were consecutively euthanized. Females developed symptoms of mild, moderate and severe HF after 10 ± 4 (*n* = 6), 18 ± 2 (*n* = 3), and 26 ± 6 weeks (*n* = 3) of RV pacing, respectively (*p* < 0.001), and were consecutively euthanized. The time of RV pacing, which induced the symptoms of particular stages of HF, was markedly shorter in males as compared to females (*p* < 0.001).

The remaining 6 males and 5 females, serving as controls, were sham-operated and followed up during 12 ± 8 and 14 ± 8 weeks (*p* > 0.2), respectively, before being euthanized.

### Longitudinal changes in echocardiography parameters in male and female pigs due to RV pacing

Sex-related differences in longitudinal changes in LVEF, LVEDV, and LA/Ao ratio were analyzed during the first 3 months of the experiment. The further analyses were not valid due to the limited number of animals surviving the later stages.

**Fig. 1:**
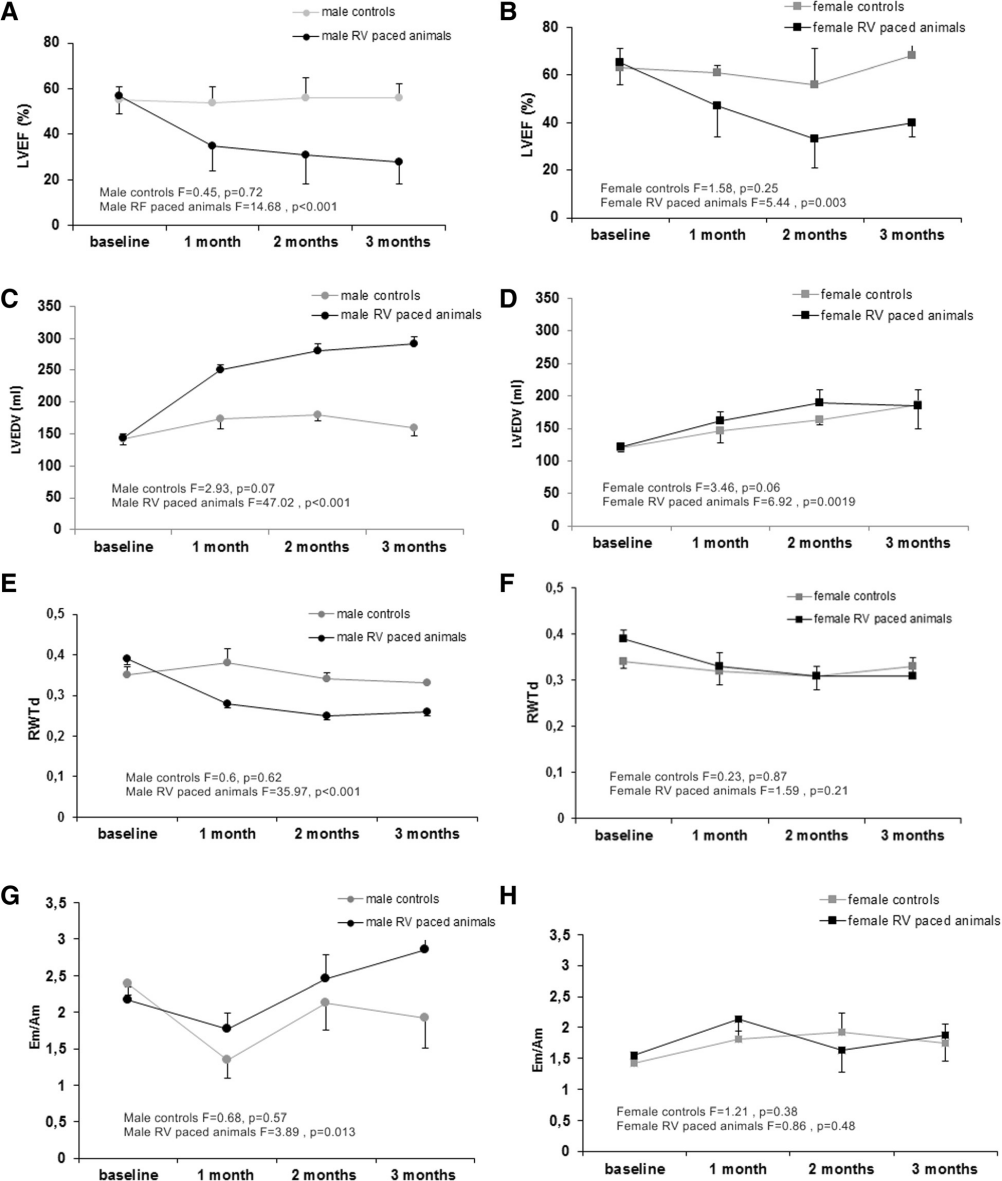
Indices of LV function at different time points for sham-operated pigs and RV-paced pigs. **a** and **b** show changes over time in left ventricular ejection fraction (LVEF, %); **c** and **d** show left ventricular end diastolic volume (LVEDV, ml); **e** and **f** show end diastolic relative wall thickness (RWTd, RWTd = 2 × LVPWd/LVIDd, *LVIDd* left ventrical internal dimension in diastole) in right ventricular (RV) paced and sham-operated pigs; **g** and **h** show the ratio of an early and late diastolic velocity (Em/Am) separately in male and female animals (*black squares*—RV paced female pigs, *black circles*—RV paced male pigs, *gray squares*—female controls, *gray circles*—male controls). Values are presented as means ± SEM. Data (separately for control and TIC animals) were tested using one-way ANOVA

The initial values (*t* = 0) for LVEF, LA/Ao ratio, LVEDV, RWTd, LVPWd, and LVPWs, SV for both sexes did not differ significantly (Additional file 1: Table S1). RV pacing resulted in gradual decline in LVEF (Fig.1a, b) and increase in the LA/Ao ratio (Additional file 1: Figure S1gh) to similar extent in both males and females. Pronounced LVEDV increase (the most intense after 1 month of pacing—68 %) was seen in male pigs, in contrast to females, where LVEDV rise was mild (Fig. 1c, d). Tachypacing-induced HF led to longitudinal decrease of RWTd (Fig. 1e, f) and LVPWd indices of LV wall remodeling only in male pigs (Additional file 1: Figure S1ab). Neither LVEF, LVEDV, RWTd, LVPWd nor LA/Ao changed in sham-operated animals of both sexes during the follow-up (Fig. 1 and Additional file 1: Figure S1).

The initial values for Em/Am differed significantly between sexes, being about 44 % higher in males (Additional file 1: Table S1). RV pacing resulted in gradual increase in the Em/Am ratio only in male pigs, whereas in male controls, as well as in control and TIC females Em/Am did not changed during the follow-up (Fig. 1g, h).

### Cross-sectional differences in echocardiography parameters measured directly before euthanasia in male and female healthy and diseased pigs

Both male and female pigs examined directly before euthanasia demonstrated reduced LVEF and increased LA/Ao along with the HF progression (all *p* < 0.05) (Tables 2 and 4). In subsequent groups of control and diseased animals, there were no sex-related differences in either LVEF or LA/Ao (*p* > 0.2) (Tables 2 and 3).

**Table 2:** Echocardiography parameters reflecting the structure and functioning of left ventricle in sham-operated male pigs (controls) and right ventricle paced pigs with induced heart failure

	LVEDV, ml	LVEDV, relative increase, %	LVEF, %	LA/Ao	RWTd	Em/Am
Controls, females	187.5 ± 35.9	115.6 ± 19.7	60.1 ± 8.4	1.43 ± 0.21	0.32 ± 0.04	1.42 ± 0.15
Mild HF, females	197.1 ± 50.5	122.9 ± 23.9	31.7 ± 12.7*	2.24 ± 0.34*	0.32 ± 0.10	2.65 ± 1.62
Moderate HF, females	273.8 ± 66.3*	122.6 ± 14.9	41.2 ± 16.9*	1.94 ± 0.49*	0.28 ± 0.04	1.91 ± 0.40
Severe HF, females	276.2 ± 104.8	99.5 ± 12.4	19.6 ± 6*	2.32 ± 0.19*	0.29 ± 0.06	2.61 ± 1.68
Controls, males	192.4 ± 24.0	120.5 ± 44.8	53.5 ± 8.4	1.38 ± 0.18	0.35 ± 0.06	1.80 ± 0.58
Mild HF, males	238.9 ± 31.1*&	148.9 ± 58.6*	42.4 ± 12.7*	1.96 ± 0.28	0.26 ± 0.05*	2.24 ± 0.87
Moderate HF, males	296.7 ± 41.8*	126.4 ± 62.0	28.0 ± 13.4*	1.98 ± 0.36*	0.27 ± 0.04*	2.51 ± 1.41
Severe HF, males	288.7 ± 34.3*&	96.0 ± 40.5	20.2 ± 6.4*	2.59 ± 0.28*	0.28 ± 0.06*	2.33 ± 0.68

*HF* heart failure, *LVEDV* left ventricular end-diastolic volume, *LVEDV, relative increase* relative increase of left ventricular end diastolic volume (LVEDV measured in end-point/LVEDV 1 month before euthanasia)*100 %; *LVEF* left ventricular ejection fraction, *RWTd* relative wall thickness at end diastole (2 × LVPWd/LVEDD, *LVEDD* left ventrical end-diastolic diameter); *LA/Ao* left atrial/aorta ratio, *Em/Am* early diastolic to late diastolic velocity ratio. All echocardiography measures were performed directly before a euthanasia. Data are presented as means ± SD

*p < 0.05 vs. control group

&*p* < 0.05 vs. corresponding female group

**Table 3:** Sex-related differences in selected echocardiography parameters and molecular markers related to myocardial remodeling assessed in left ventricle in male and female pigs with and without tachycardia-induced cardiomyopathy (the results of the two-way ANOVA)

Variables (units)	Sex	HF groups	Interactions (sex and HF groups)
	F p	F p	F p
LVEF (%)	0.85 0.36	9.59 < 0.001	1.06 0.38
LVEDV (ml)	6.61 0.02	8.04 < 0.001	2.51 0.08
LVEDV, relative increase (%)	0.26 0.61	3.83 0.019	1.74 0.18
LA/Ao	0.00 0.95	14.79 < 0.001	1.71 0.18
RWTd	0.94 0.34	3.59 0.02	0.64 0.59
Em/Am	0.11 0.74	0.20 0.89	0.25 0.86
BNP, mRNA (AU)	7.16 0.01	3.36 0.03	2.07 0.12
GATA4, mRNA (AU)	5.29 0.03	1.22 0.32	1.04 0.39
TGF-β1, mRNA (AU)	3.43 0.07	0.88 0.46	0.70 0.56
MMP9, mRNA (AU)	0.06 0.81	2.49 0.08	1.81 0.16
TIMP1, mRNA (AU)	0.68 0.42	1.59 0.21	0.74 0.53
NGAL, mRNA (AU)	4.65 0.04	1.00 0.40	1.65 0.20
Col1A1, mRNA (AU)	3.54 0.07	2.67 0.06	0.72 0.55
Col1A2, mRNA (AU)	5.43 0.03	2.51 0.08	1.42 0.26
Col3A1, mRNA (AU)	0.22 0.65	3.68 0.02	0.48 0.70
Water soluble collagen (μg/mg wet tissue)	5.22 0.03	2.59 0.08	0.76 0.53
Total recoverable collagen (μg/mg wet tissue)	0.63 0.43	0.78 0.51	1.59 0.21
Total gelatinolytic activity (AU)	15.59 0.001	1.02 0.40	0.46 0.71
Cross-sectional cardiomyocyte diameter (μm)	5.84 0.02	0.17 0.92	1.78 0.34

*LVEF* left ventricle ejection fraction, *LVEDV* left ventricle end-diastolic volume, *LVEDV, relative increase* LVEDV in end-point/LVEDV 1 month before euthanasia)*100 %, *LA/Ao* left atrial/aorta ratio, *RWTd* relative wall thickness at end diastole (2 × LVPWd/LVEDD, *LVPWd* end-diastolic thickness of left ventricle posterior wall, *LVEDD* left ventrical end-diastolic diameter), *Em/Am* the ratio of an early and late diastolic velocity, *BNP* B-type natriuretic peptide, *GATA4* GATA binding protein 4, *TGF-β1* transforming growth factor β1, *MMP9* matrix metalloproteinase type 9, *NGAL* neutrophil gelatinase-associated lipocalin, *TIMP1* tissue inhibitor of metalloproteinases type 1, *Col1A1* collagen, type I, alpha 1, *Col1A2* collagen type I, alpha 2, *Col3A1* collagen type III, alpha 1

There were no differences in LVEDV measured directly before euthanasia between healthy and diseased female pigs (Table 4). In contrast, LVEDV was higher in male HF pigs as compared to controls (*p* < 0.001) (Table 2). Data analysis revealed that LVEDV is a sex-dependent parameter (Tables 3 and 4). To follow the dynamic of LV dilatation, we calculated the relative increase of LVEDV in last month before euthanasia [(LVEDV in end-point/LVEDV 1 month before euthanasia)*100 %]. The process of LV dilatation seemed to be most pronounced in males from the mild HF group (Table 2). What is more, HF development in males was accompanied by RWTd decrease (Tables 2, 3, and 4); however, no signs of LV thinning were evidenced (Additional file 1: Tables S2, S3 and S4).

**Table 4:** Relationships between selected echocardiographic parameters and molecular markers related to myocardial remodelling assessed in left ventricle and HF in male and female pigs with and without tachycardia-induced cardiomyopathy (the results of Spearman’s rank correlation coefficients)

Variables (units)	Male		Female	
	*r*	*p*	*r*	*p*
LVEF (%)	−0.76	<0.001	−0.54	0.03
LVEDV (ml)	0.76	<0.001	0.18	0.50
LVEDV, relative increase (%)	−0.42	0.04	−0.2	0.47
LA/Ao	0.78	<0.001	0.62	0.009
RWTd	−0.56	0.002	−0.23	0.38
Em/Am	0.20	0.35	−0.07	0.78
BNP, mRNA (AU)	0.74	<0.001	0.75	<0.001
GATA4, mRNA (AU)	0.63	0.004	0.49	0.06
TGF-β1, mRNA (AU)	0.61	0.001	0.13	0.63
MMP9, mRNA (AU)	−0.08	0.71	0.00	0.99
TIMP1, mRNA (AU)	−0.04	0.84	0.17	0.54
NGAL, mRNA (AU)	−0.59	0.002	0.16	0.54
Col1A1, mRNA (AU)	0.31	0.14	−0.23	0.41
Col1A2, mRNA (AU)	0.20	0.33	−0.33	0.23
Col3A1, mRNA (AU)	0.09	0.65	−0.24	0.41
Water soluble collagen (μg/mg wet tissue)	−0.41	0.06	0.06	0.85
Total recoverable collagen (μg/mg wet tissue)	−0.16	0.44	−0.19	0.53
Total gelatinolytic activity (AU)	−0.49	0.02	−0.32	0.31
Cross-sectional cardiomyocyte diameter (μm)	−0.36	0.09	0.53	0.08

*LVEF* left ventricle ejection fraction, *LVEDV* left ventricle end-diastolic volume, *LVEDV, relative increase* (LVEDV in end-point/LVEDV 1 month before euthanasia)*100 %, *LA/Ao* left atrial/aorta ratio, *RWTd* relative wall thickness at end diastole (2 × LVPWd/LVEDD, *LVPWd* end-diastolic thickness of left ventricle posterior wall, *LVEDD* left ventrical end-diastolic diameter), *Em/Am* the ratio of an early and late diastolic velocity, *BNP* B-type natriuretic peptide, *GATA4* GATA binding protein 4, *TGF-β1* transforming growth factor β1, *MMP9* matrix metalloproteinase type 9, *NGAL* neutrophil gelatinase-associated lipocalin, *TIMP1* tissue inhibitor of metalloproteinases type 1, *Col1A1* collagen, type I, alpha 1, *Col1A2* collagen type I, alpha 2, *Col3A1* collagen type III, alpha 1

### mRNA expression of selected genes related to neurohormonal activation, hypertrophy, and extracellular matrix-related in LV myocardium in male and female healthy and diseased pigs

**Fig. 2:**
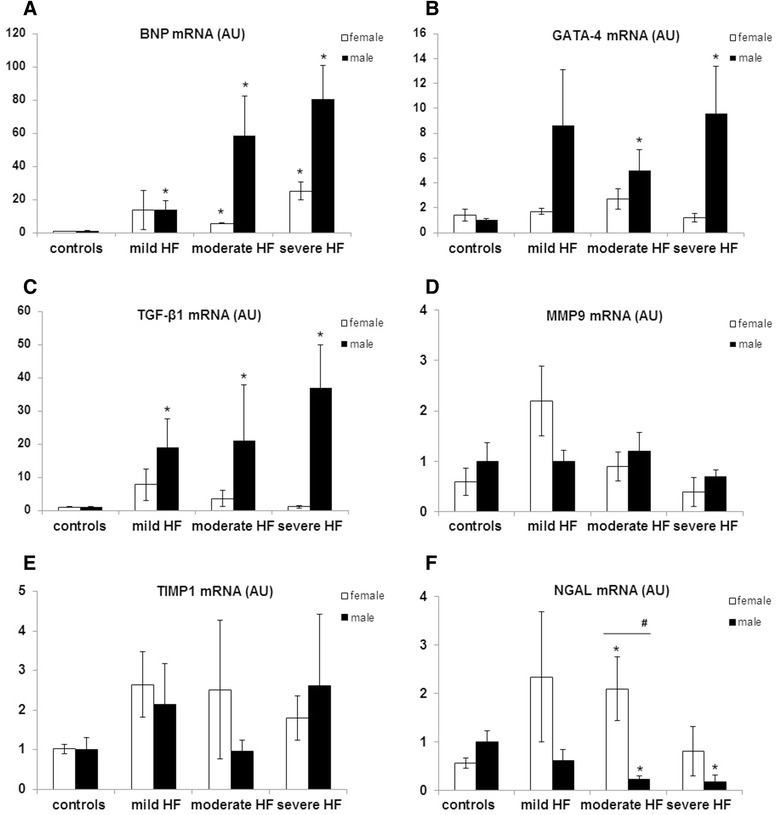
RNA expression of selected genes related to neurohormonal activation, hypertrophy, and extracellular matrix remodeling. RNA level was determined in left ventricle myocardium in sham-operated pigs (controls) and in right ventricle (RV) paced pigs in subsequent stages of heart failure (mild, moderate, and severe HF), separately in male (*black columns*) and female animals (*white columns*). **a** B-type natriuretic peptide (BNP), **b** GATA binding protein 4 (GATA4), **c** transforming growth factor-β1 (TGF-β1), **d** matrix metalloproteinase type 9 (MMP9), **e** tissue inhibitor of metalloproteinases type 1 (TIMP1), and **f** neutrophil gelatinase-associated lipocalin (NGAL). mRNA expression was quantified using real-time RT-PCR and normalized against the product of GAPDH. Values are presented as means ± SEM. *p < 0.05 vs. the control group; ^**#**^ *p* < 0.05 female vs. male pigs (in particular study group)

The progression of HF was associated with a marked increase in BNP mRNA expression in LV myocardium in both male and female RV paced pigs, as compared to sham-operated animals (both *p* < 0.001) (Table 4, Fig. 2a). However, the BNP mRNA expression in LV myocardium was higher in male than female pigs with HF (*p* = 0.01) (Table 3). The increased BNP expression in male LV myocardium was accompanied by LV dilatation (LVEDV: *r* = 0.65, *p* < 0.001) and systolic dysfunction (LVEF: *r* = −0.66, *p* < 0.001) whereas in females, only by systolic dysfunction (LVEF: *r* = −0.66, *p* < 0.001) (Additional file 1: Figure S2).

The development of HF was accompanied by steady increase in mRNA expression of both GATA4 and TGF-β1 in LV myocardium in male pigs (both *p* < 0.01) (Table 2, Fig. 2b, c). Males had a consistently greater expression of GATA4 (*p* = 0.03) and tendency for TGF-β1 mRNA level to increase (borderline *p* = 0.07) in LV myocardium as compared to females in consecutive groups of diseased animals (Table 3). The levels of GATA4 mRNA correlated with BNP mRNA (males: *r* = 0.63, *p* = 0.005; females: *r* = 0.66, *p* = 0.007) (Additional file 1: Figure S3). The GATA4 expression was correlated with the level of TGF-β1 mRNA (*r* = 0.83, *p* < 0.001), as well as with systolic dysfunction indices (*r* = −0.68, *p* = 0.002) only in males (Additional file 1: Figure S3).

**Fig. 3:**
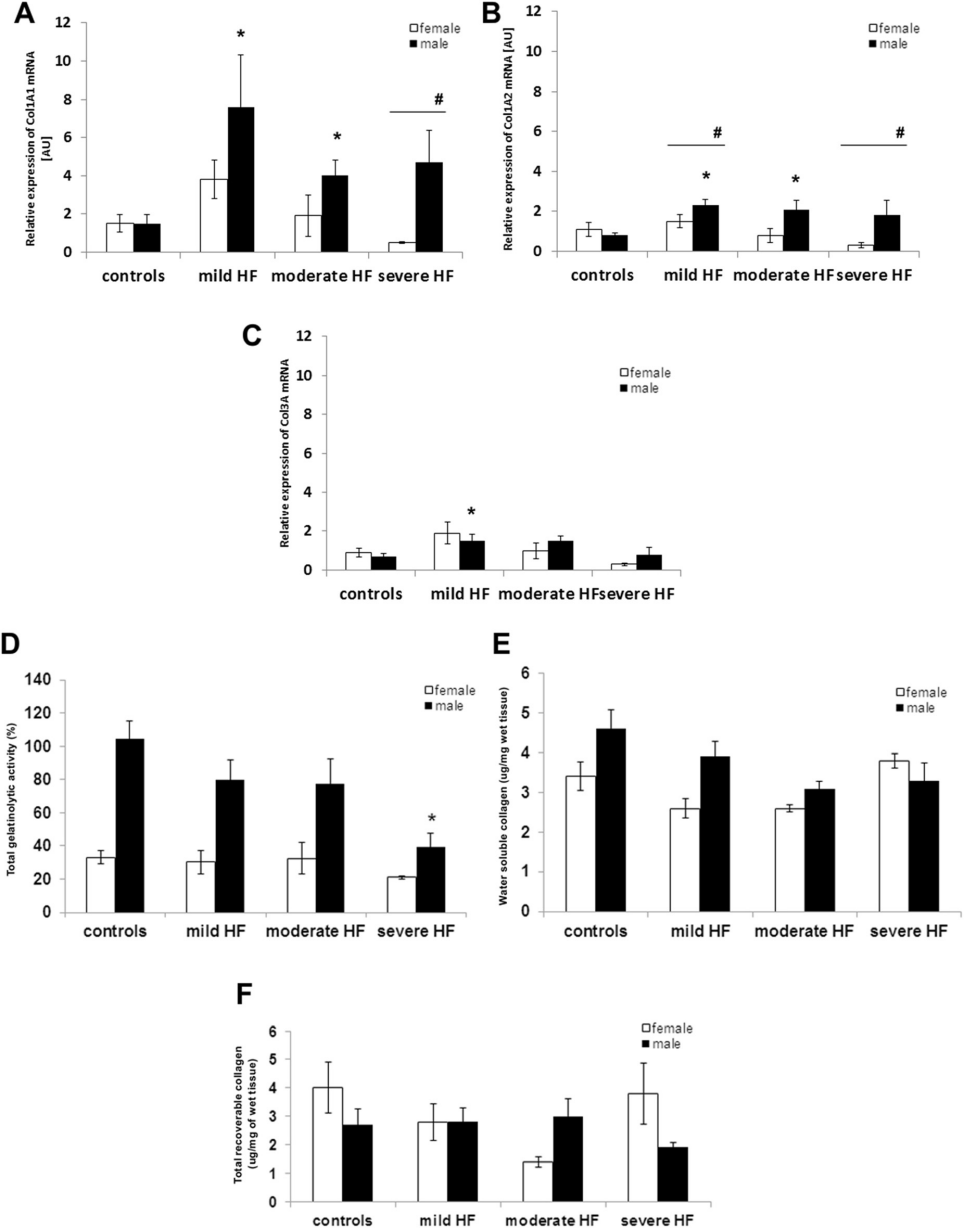
Extracellular matrix turnover indices in sham-operated pigs and in right ventricle (RV) paced pigs. Collagen mRNA and ECM turnover was evaluated in subsequent stages of heart failure (mild, moderate, and severe HF), separately in male (*black columns*) and female animals (*white columns*). **a** Col1A1—collagen, type I, alpha 1; **b** Col1A2—collagen type I, alpha 2; **c**—Col3A1collagen type III, alpha 1. **d** shows total gelatinolytic activity quantified using an biotin-gelatin assay; **e** shows water soluble collagen (μg/mg of wet tissue); **f** shows total recoverable collagen (μg/mg of wet tissue); Values are presented as means ± SEM. *p < 0.05 vs. the control group; ^**#**^ *p* < 0.05 female vs. male pigs (in particular study group)

There were no differences in the mRNA expression of MMP9 and TIMP1 in LV myocardium across all the examined groups of both sexes (Tables 3 and 4, Figs. 2d and 3e). However, the progression of HF was associated with a reduced NGAL mRNA expression in LV myocardium only in male pigs (all *p* < 0.01) (Tables 3 and 4, Fig. 2f). A decrease of the NGAL expression in males was accompanied by a LV dilatation (*r* = −0.52, *p* = 0.008) (Additional file 1: Figure S4).

**Fig. 4:**
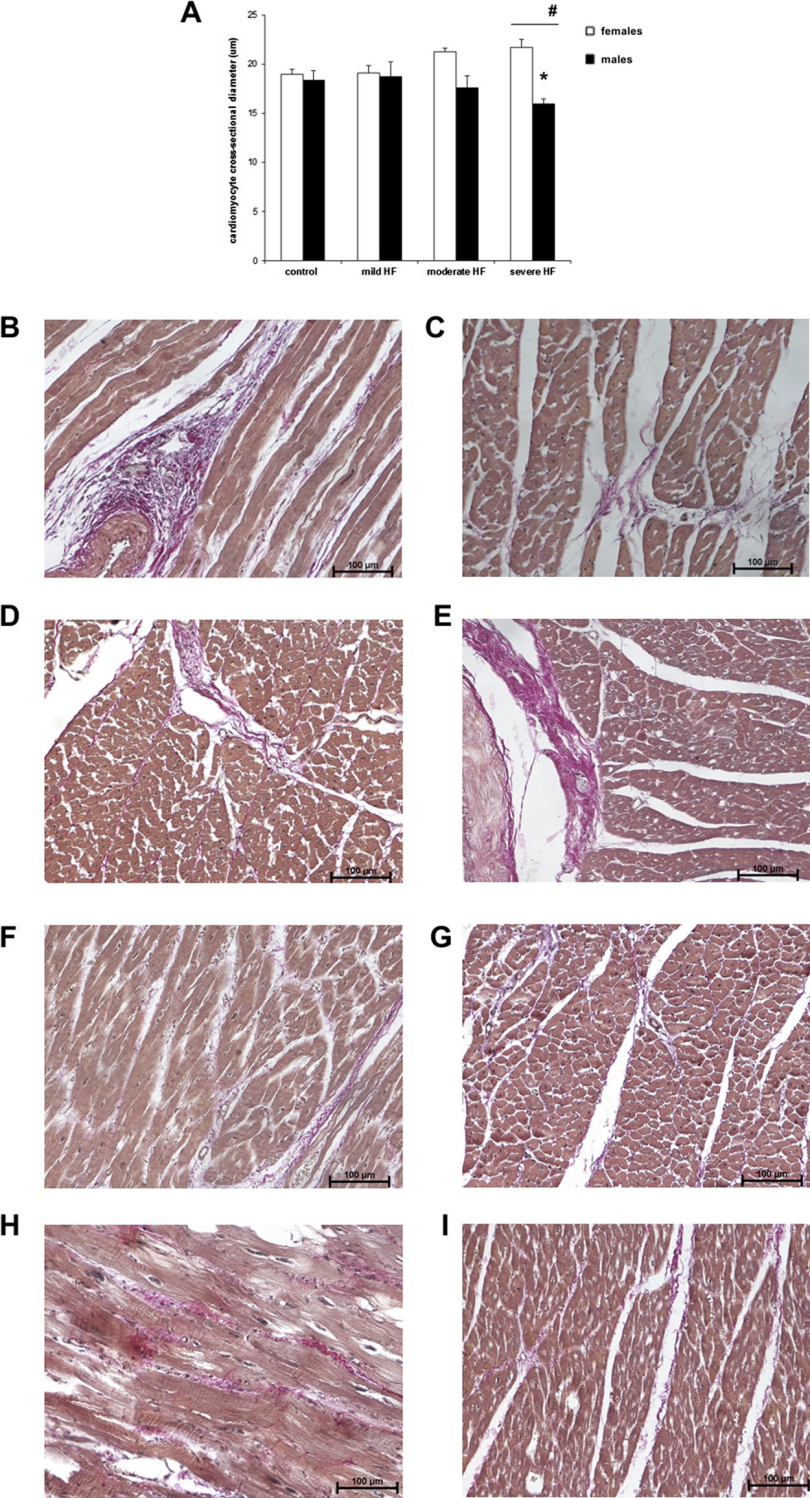
Histological analysis of cardiomyocyte cross-sectional diameter and collagen content in LV myocardium. **a** represents cardiomyocyte cross-sectional diameter in subsequent stages of heart failure (mild, moderate, and severe HF), separately in male (*black columns*) and female animals (w*hite columns*). Values are presented as means ± SEM. *p < 0.05 vs. the control group; ^**#**^ *p* < 0.05 female vs. male pigs (in particular study group). Representative photomicrograph of van Gieson-stained sections of LV demonstrating interstitial collagen content and myocyte cross-sectional diameter in controls (sham-operated, **b** female, **c** male) and in subsequent stages of heart failure in females (**d** mild, **f** moderate, and **h** severe HF) and in males (**e** mild, **g** moderate, and **i** severe HF). Original magnification ×200; *scale bar*—100 μm). Note that collagenous network (*red*) surrounding muscle bundles (*yellow*) is similar in all shown sections

In consecutive groups of male pigs, higher expression of Col1A2 mRNA and borderline Col1A1 (but not Col3A1) was consistently observed as compared to females (Table 2, Fig. 4a-c). Col3A1 mRNA level was associated with HF development, regardless of sex (Tables 3 and 4). In both sexes, collagen mRNA levels correlated with the level of TGF-β1 mRNA (Additional file 1: Figure S5).

### Total gelatinolytic activity and the amount of water soluble and total recoverable collagen in LV myocardium in male and female healthy and diseased pigs

Total gelatinolytic activity in LV myocardium was higher in male than female pigs (both with and without HF, *p* < 0.05), and the progression of HF was associated with reduced total gelatinolytic activity (*p* < 0.05). A tendency for water soluble collagen to decrease (borderline, *p* = 0.06) in LV myocardium was observed only in male pigs (Tables 3 and 4, Fig. 3d, e).

The decrease of total gelatinolytic activity in male pigs was accompanied by a BNP mRNA upregulation (*r* = −0.48, *p* = 0.03). Decrease of water soluble collagen correlated with NGAL mRNA (*r* = 0.52, *p* = 0.009) (Additional file 1: Figure S6). In male subjects from mild HF group, the water soluble collagen correlated with relative increase in LVEDV (*r* = 0.78, *p* = 0.036) (Additional file 1: Figure S6). There were no differences in the amount of total recoverable collagen in the LV myocardium across all examined groups of both sexes (Tables 3 and 4, Fig. 3f).

### Volume fraction of myocardial collagen and myocyte diameter

Assessment of van Gieson-stained LV tissue sections using conventional light microscopy revealed that collagenous network surrounding muscle bundles was similar in controls and HF animals, irrespective of sex (Fig. 4). The collagen content of the myocardium was histologically quantified as the nonvascular myocardial area occupied by collagen (% tissue section area). No differences in collagen content were found between HF groups and sexes. The collagen content of the myocardium did not correlate with total recoverable collagen.

Cardiomyocyte cross-sectional diameter was quantified from van Gieson-stained sections. The cardiomyocyte diameter was significantly smaller in male hearts as compared to females (Table 3). What is more, males with severe HF have decreased cardiomyocyte diameter as compared to controls and females in relevant HF groups (Fig. 4a). In females, cardiomyocyte diameter correlated with LVEDV (*r* = 0.74, *p* = 0.05) (Additional file 1: Figure S7), what was not observed in males.

## Discussion

We compared the echocardiography and molecular measures of myocardial remodeling in male and female pigs with chronic symptomatic systolic HF due to RV pacing. The HF progression was associated with a similar reduction in LVEF as well as an increase in the left atrium diameter in both genders. Male subjects, characterized by significantly higher gelatinolytic activity in the myocardium, developed LV dilatation, which was accompanied by the pro-hypertrophic gene program induction. In contrast, HF progression in female pigs was not associated with intense LV remodeling.

Although sex differences in HF prevalence and severity have long been recognized [1], the molecular mechanisms responsible for them are poorly understood. Literature regarding this problem is scarce and limited to the rodent models of HF [7–10]. To our knowledge, our report is the first to address sex differences in HF development on a large animal model. In our model, similar to human and rodent studies [33, 34], males demonstrated earlier transition into HF than female counterparts [8]. There were no sex differences in systolic dysfunction and left atrial size increase along with the HF development. In turn, males demonstrated significant LV dilatation, whereas females did not. This is consistent with observations made on sex-related differences in human cardiac remodeling [6].

Cardiac remodeling during HF progression is a dynamic process, which involves both cardiomyocytes and ECM. ECM provides a structural support network that helps to maintain myocardial architecture [35]. In normal myocardium, the composition and orientation of ECM is tightly regulated; the deposition and degradation of matrix components represent a well-balanced equilibrium [36]. Matrix metalloproteinases (MMPs) play an important role in this process [35], digesting collagens, the most abundant proteins forming ECM [36]. To better understand the sex-related differences in LV remodeling, some indices of the myocardial ECM turnover were analyzed. As fibrillar collagen types I and III coexist to form the collagenous network of the myocardium comprising 85 and 11 % of the collagen in healthy mammalian hearts, respectively [36], we analyzed the expression of both collagen types. Although male HF pigs had higher levels of collagen I mRNA in consecutive groups of animals and collagen III mRNA level correlated with HF development (regardless of sex), these differences seem to not result in the collagen accumulation within the myocardium. While mRNA level of fibrillar collagen can be used as index of the directionality of ECM remodeling, this measurement alone is not conclusive [37]. Specifically, processing of the newly secreted procollagen molecules is essential for the formation of stable fibrils and collagen accumulation within the myocardium [37]. Mature collagen appears to be almost completely inert, with about 1000 days half-life [38]. Thus, it is likely that contribution of newly synthesized collagen may be too low to impact the measure of total myocardial collagen. On the other side, loss of interstitial collagen was not noted in our model, in contrast to results by Spinale et al. [39] reporting that the collagen weave was markedly reduced and disrupted in tachypacing heart failure in pigs. However, this TIC model was developed in young, 20–30 kg weight piglets using rapid pacing at 220–240 bpm up to 3 weeks, and resulted in the development of acute or subacute rather than chronic HF [39]. RV tachypacing in young and aged sheep led to collagen accumulation in young animals and collagen depletion in aged animals parallel to the symptoms of cardiac decompensation [40]. Lack of significant alteration in collagen content along with HF development observed in our model does not rule out its damage as the collagen network is extensively cross-linked. Cleavage of collagen fibers may result in dysfunction of the collagen network, but release of collagen degradation products can be difficult to observe [41]. To test this assumption, we analyzed the total gelatynolytic activity in LV myocardial samples. Due to its properties (self-aggregation and crosslinking), collagens can be degraded in vivo only by concerted action of MMPs [24, 42]. Firstly, the helix is cleaved about three quarters of the way from the N-terminus by interstitial MMPs (i.e., MMP1, [43]) and then further degradation to small fragments is performed by gelatinases (MMP2 and MMP9) [24]. The use of biotinylated gelatin as a substrate allowed us to measure a net gelatynolytic activity in myocardial samples which is more physiologically relevant than the total amount of activatable enzyme or immunoreactive material [44]. Contrary to our initial hypotheses, we did not observe any increase in total gelatynolytic activity in LV myocardial samples during HF development. Moreover, total gelatinolytic activity in LV myocardium was higher in male than female pigs (irrespective of HF development). Also, the amount of water soluble collagen (low molecular weight products of MMPs activity, containing collagen fragments small enough to dissolve in water, but able to bind Sirius red stain) was higher in male LV homogenates. This may suggest that male myocardium has an increased ECM degradation potential. This could be one of the reasons for different adaptation to RV pacing observed in male and female porcine hearts. While male LV initially dilated fast following pacing (68 % LVEDV increase in first month), female LVEDV remained unchanged. Thus, porcine LV response to increased preload seems to be sex-related. ECM myocardial remodeling in males starts earlier than in females, probably due to higher gelatinolytic activity per se. In a rodent model of MI, increased myocardial MMPs activity in males was related to enhanced maladaptive remodeling [9, 45]. The analysis of factors related to ECM remodeling in our TIC model has shown the NGAL level to be sex-specific. Its expression was higher in males and correlated with the products of collagen degradation, suggesting a possible role of this protein in enhanced ECM degradation. NGAL was shown to protect MMP9 from degradation, and thereby preserving its enzymatic activity [46]. Relevant data from human or large animal models are scarce. The only available data concern PO-induced LV hypertrophy due to aortic stenosis [3]. Women and men with aortic stenosis differ in their ECM-related gene expression; maladaptive LV remodeling occurs more frequently in men and is associated with a greater activation of ECM-related genes [3].

Based on our observations, we can hypothesize that, due to higher myocardial gelatynolytic activity, there is more partially cleaved collagen in males than in females, making the collagen network more flexible in male hearts. Thus, TIC-induced increased preload rapidly dilates male LV in the first month of pacing. Moreover, expansion of the ECM scaffold in males led to the eccentric LV remodeling, accompanied by significant change of cardiomyocyte cross-sectional diameter. These long slender myocytes may also be at a mechanical disadvantage due to inadequate adaptive growth of the myocyte cross-sectional area responsible for force generation [47]. In contrast, female LV chambers seem to be stiffer (initial Em/Am ratio was about 60 % lower than in males), probably partially due to the lower myocardial gelatinolytic activity. In females, the LV dilated much slower, without pronounced signs of eccentric hypertrophy. Female hearts preserved initial diastolic function and remained relatively stiff along with the HF development. This observation is relevant to the fact that women are considered more likely than men to have LV diastolic dysfunction as the main underlying pathophysiological abnormality of HF [48]. Heart failure with normal ejection fraction (HF-NEF) is believed to be more common in women than in men [49]. Clinically HF-NEF is characterized by the symptoms and signs of HF in the presence of a normal left ventricular ejection fraction and diastolic LV dysfunction evident from slow LV relaxation and high LV stiffness [50].

The sex-related differences in pigs were also mirrored by the LV gene expression pattern of hypertrophy-related elements. An increase in the size of LV cavity related to a hemodynamic overload and ECM dissolution results in biomechanical wall stretch stress [51] and induces GATA4 expression [52]. GATA4 is a crucial regulator of adaptive cardiac growth in response to pathologic stimulation and acts by activating fetal genes [53]. GATA4 induction is known to be followed by increase in the BNP expression [52, 54]. As expected, the induction of the fetal gene program was noted only in male pigs. What is more, TGF-β1, a crucial factor promoting myocardial hypertrophy [55], was upregulated in the LV from males but not from females following experimental HF development. A similar observation was made in a rodent pressure overload (PO) model [56]. Data on sex-related differences in the LV gene expression patterns of myocardial hypertrophy-related elements accompanying HF in large animals and humans is scarce. Women and men with aortic stenosis differ in terms of their hypertrophy-related gene expression; TGF-β1 signaling pathways were shown to be activated only in male patients [57].

A physiological, exercise-like phenotype of myocardial hypertrophy with maintained cardiac function without fibrosis predominates in females [58]. Cardiovascular changes that occur in pregnancy represent one of the forms of physiological hypertrophy and resemble those found in exercise training [59]. In pregnancy, the female heart increases its cardiac output and adapts to an increasing cardiac load without significant fibrosis in a fully reversible manner [58]. We can speculate that a low LV ECM turnover in female pigs may be useful in gradual LV adaptation to an increased preload and protects the LV from dilatation.

Our findings, showing sex differences in some aspects of the ECM turnover leading to a diverse myocardial response to RV pacing, highlight the need for sex-specific medicine. While it is women who predominantly die of cardiovascular diseases, they are markedly underrepresented in clinical studies [60]. What is more, care should be taken in analyzing the results from animal HF models as some effects might be sex-specific.

This work has obviously some limitations. Although the number of experimental animals being investigated may seem low, it is nevertheless in good line with studies using similar pig models [16, 17]. In contrast to rodent experimental models, housing and maintenance costs for large animals are significantly higher. However, porcine hearts, exhibiting a gross anatomic structure very similar to that of humans, seem to be suitable for translational studies [12]. Rapid RV pacing in pigs has been shown to produce a progressive, reliable model of dilated cardiomyopathy (DCM) and chronic HF [61]. We believe that our results may be helpful in finding new therapeutic approaches specifically tailored to adverse ECM remodeling in human HF.

## Conclusions

The present study demonstrates that porcine male and female hearts respond differently to chronic right ventricular pacing. Males demonstrated significant LV dilatation, followed by a strong induction of pro-hypertrophic gene program, and an earlier development of symptomatic HF

## Additional file

Additional file 1: Figure S1. Indices of LV function at different time points for sham-operated pigs and RV-paced pigs. A and B show changes over time in LVPWd (LVPWd—end-diastolic thickness of left ventricle posterior wall); C and D show LVPWs—end-systolic thickness of left ventricle posterior wall, E and F show SV (stroke volume) in right ventricular (RV) paced and sham-operated pigs; G and H show LA/Ao (left atrial/aorta ratio) separately in male and female animals (black squares—RV paced female pigs, black circles—RV paced male pigs, gray squares—female controls, gray circles—male controls). Values are presented as means ± SEM. Data (separately for control and TIC animals) were tested using one-way ANOVA. **Figure S2**. Relationship between BNP mRNA expression and echocardiographic parameters. Elevated BNP mRNA was significantly correlated with increased LVEDV in males (A) as well as with a decrease in LVEF in males (B) and females (C). Regression lines have been fit to all data points. **Figure S3**. Relationship between GATA4 mRNA expression and TGFβ1 and BNP expression level as well as LVEF. Elevated GATA4 mRNA was significantly correlated with increased BNP mRNA level in males (A) as well as in females (B). Only in males elevated GATA4 mRNA was also significantly correlated with TGFβ1 BNP mRNA level (C) and with a decrease in LVEF (D). Regression lines have been fit to all data points. **Figure S4**. Relationship between NGAL mRNA expression and LVEDV. Elevated NGAL mRNA was significantly correlated with increased LVEDV in males. Regression lines have been fit to all data points. **Figure S5**. Relationship between collagen mRNA level and TGFβ1 expression. Elevated Col1A1 (A), Col1A2 (B), and Col3A1 (C) mRNAs were significantly correlated with an increase in TGFβ1 mRNA level in males. In females increased expression of Col1A1 (D) and Col3A1 (E) was related to TGFβ1 mRNA level. Regression lines have been fit to all data points. **Figure S6**. Relationship between ECM turnover markers and echocardiographic parameters. (A) Reduced total gelatinolytic activity was significantly correlated with an increase in BNP mRNA level in males. (B) Decreasing water soluble collagen was related to an decrease in NGAL mRNA. (C) In male subjects from the mild HF group, the water soluble collagen correlated with the relative increase in LVEDV. Regression lines have been fit to all data points. **Figure S7**. Relationship between cardiomyocyte diameter and echocardiographic parameters. In females cardiomyocyte cross-sectional diameter correlated with LVEDV. Regression lines have been fit to all data points. **Table S1**. Initial values of echocardiography parameters in sham-operated male pigs (controls) and right ventricle paced pigs with induced heart failure. **Table S2**. Echocardiography parameters reflecting the structure and functioning of left ventricle in sham-operated male pigs (controls) and right ventricle paced pigs with induced heart failure. **Table S3**. Sex-related differences in selected echocardiography parameters assessed in left ventricular myocardium in male pigs with and without tachycardia-induced cardiomyopathy (the results of the two-way ANOVA). **Table S4**. Relationships between selected echocardiographic parameters and HF insufficiency in male and female pigs with and without tachycardia-induced cardiomyopathy (the results of Spearman’s rank correlatory coefficients).

## Abbreviations

BNP: B-type natriuretic peptide
Col1A1: collagen, type I, alpha 1
Col1A2: collagen type I, alpha 2
Col3A1: collagen type III, alpha 1
ECG: echocardiography
bpm: beat per minute
ECM: extracellular matrix
Em/Am: early diastolic to late diastolic velocity ratio
GAPDH: glyceraldehyde-3-phosphate dehydrogenase
GATA4: GATA binding protein 4
HF: heart failure
LA/Ao: ratio of the left atrium to the diameter of the aorta
LV: left ventricle
LVEDD: LV end diastolic diameter
LVEDV: LV end-diastolic volume
LVEF: LV ejection fraction
LVPWd: end-diastolic thickness of left ventricle posterior wall
LVPWs: end-systolic thickness of left ventricle posterior wall
LVESV: LV end-systolic volume
MMP: matrix metalloproteinase
NGAL: neutrophil gelatinase-associated lipocalin
RWTd: relative wall thickness at end diastole (2 × LVPWd/LVEDD, LVEDD—left ventrical end-diastolic diameter)
RV: right ventricle
SV: stroke volume
TGF-β1: tissue growth factor β1
TIC: tachycardia-induced cardiomyopathy
TIMP1: tissue inhibitor of metalloproteinases type 1
