# Supplementary material for: Sex differences in porcine left ventricular myocardial remodeling due to right ventricular pacing
Source: Biol Sex Differ. 2015 Dec 10;6:32. doi: 10.1186/s13293-015-0048-4 (PMC4676102; doi:10.1186/s13293-015-0048-4)
Supplement: Additional file 1: Figure S1. — Indices of LV function at different time points for sham-operated pigs and RV-paced pigs. A and B show changes over time in LVPWd (LVPWd—end-diastolic thickness of left ventricle posterior wall); C and D show LVPWs—end-systolic thickness of left ventricle posterior wall, E and F show SV (stroke volume) in right ventricular (RV) paced and sham-operated pigs; G and H show LA/Ao (left atrial/aorta ratio) separately in male and female animals (black squares—RV paced female pigs, black circles—RV paced male pigs, gray squares—female controls, gray circles—male controls). Values are presented as means ± SEM. Data (separately for control and TIC animals) were tested using one-way ANOVA. Figure S2. Relationship between BNP mRNA expression and echocardiographic parameters. Elevated BNP mRNA was significantly correlated with increased LVEDV in males (A) as well as with a decrease in LVEF in males (B) and females (C). Regression lines have been fit to all data points. Figure S3. Relationship between GATA4 mRNA expression and TGFβ1 and BNP expression level as well as LVEF. Elevated GATA4 mRNA was significantly correlated with increased BNP mRNA level in males (A) as well as in females (B). Only in males elevated GATA4 mRNA was also significantly correlated with TGFβ1 BNP mRNA level (C) and with a decrease in LVEF (D). Regression lines have been fit to all data points. Figure S4. Relationship between NGAL mRNA expression and LVEDV. Elevated NGAL mRNA was significantly correlated with increased LVEDV in males. Regression lines have been fit to all data points. Figure S5. Relationship between collagen mRNA level and TGFβ1 expression. Elevated Col1A1 (A), Col1A2 (B), and Col3A1 (C) mRNAs were significantly correlated with an increase in TGFβ1 mRNA level in males. In females increased expression of Col1A1 (D) and Col3A1 (E) was related to TGFβ1 mRNA level. Regression lines have been fit to all data points. Figure S6. Relationship between ECM turnover markers and echocardiographic pa [file 13293_2015_48_MOESM1_ESM.pdf]

Figure S1 Indices of LV function at different time points for sham-operated pigs and RV-paced pigs. A and **B** show changes over time in LVPWd (LVPWd – end-diastolic thickness of left ventricle posterior wall); **C** and **D** show LVPWs – end-systolic thickness of left ventricle posterior wall, **E** and **F** show SV (stroke volume) in right ventricular (RV) paced and sham-operated pigs; **G** and **H** show LA/Ao (left atrial/aorta ratio) separately in male and female animals (black squares – RV paced female pigs, black circles – RV paced male pigs, gray squares – female controls, gray circles – male controls). Values are presented as means±SEM. Data (separately for control and TIC animals) were tested using one-way ANOVA.

Figure S2 Relationship between BNP mRNA expression and echocardiographic parameters. Elevated BNP mRNA was significantly correlated with increased LVEDV in males (**A)** as well as with a decrease in LVEF in males (**B**) and females (**C**). Regression lines have been fit to all data points.

Figure S3 Relationship between GATA4 mRNA expression and TGFβ1 and BNP expression level as well as LVEF. Elevated GATA4 mRNA was significantly correlated with increased BNP mRNA level in males (**A)** as well as in females **(B).** Only in males elevated GATA4 mRNA was also significantly correlated with TGFβ1 BNP mRNA level (**C**) and with a decrease in LVEF (**D**). Regression lines have been fit to all data points.

Figure S4 Relationship between NGAL mRNA expression and LVEDV. Elevated NGAL mRNA was significantly correlated with increased LVEDV in males. Regression lines have been fit to all data points.

Figure S5 Relationship between collagen mRNA level and TGFβ1 expression. Elevated Col1A1 (**A**), Col1A2 (**B**) and Col3A1 (**C**) mRNAs were significantly correlated with an increase in TGFβ1 mRNA level in males. In females increased expression of Col1A1 **(D)** and Col3A1 **(E)** was related to TGFβ1 mRNA level. Regression lines have been fit to all data points.

Figure S6 Relationship between ECM turnover markers and echocardiographic parameters. (**A**) Reduced total gelatinolytic activity was significantly correlated with an increase in BNP mRNA level in males. (**B**) Decreasing water soluble collagen was related to an decrease in

NGAL mRNA**. (C)** In male subjects from the mild HF group, the water soluble collagen correlated with the relative increase in LVEDV. Regression lines have been fit to all data points.

Figure S7 Relationship between cardiomyocyte diameter and echocardiographic parameters. In females cardiomyocyte cross-sectional diameter correlated with LVEDV. Regression lines have been fit to all data points.

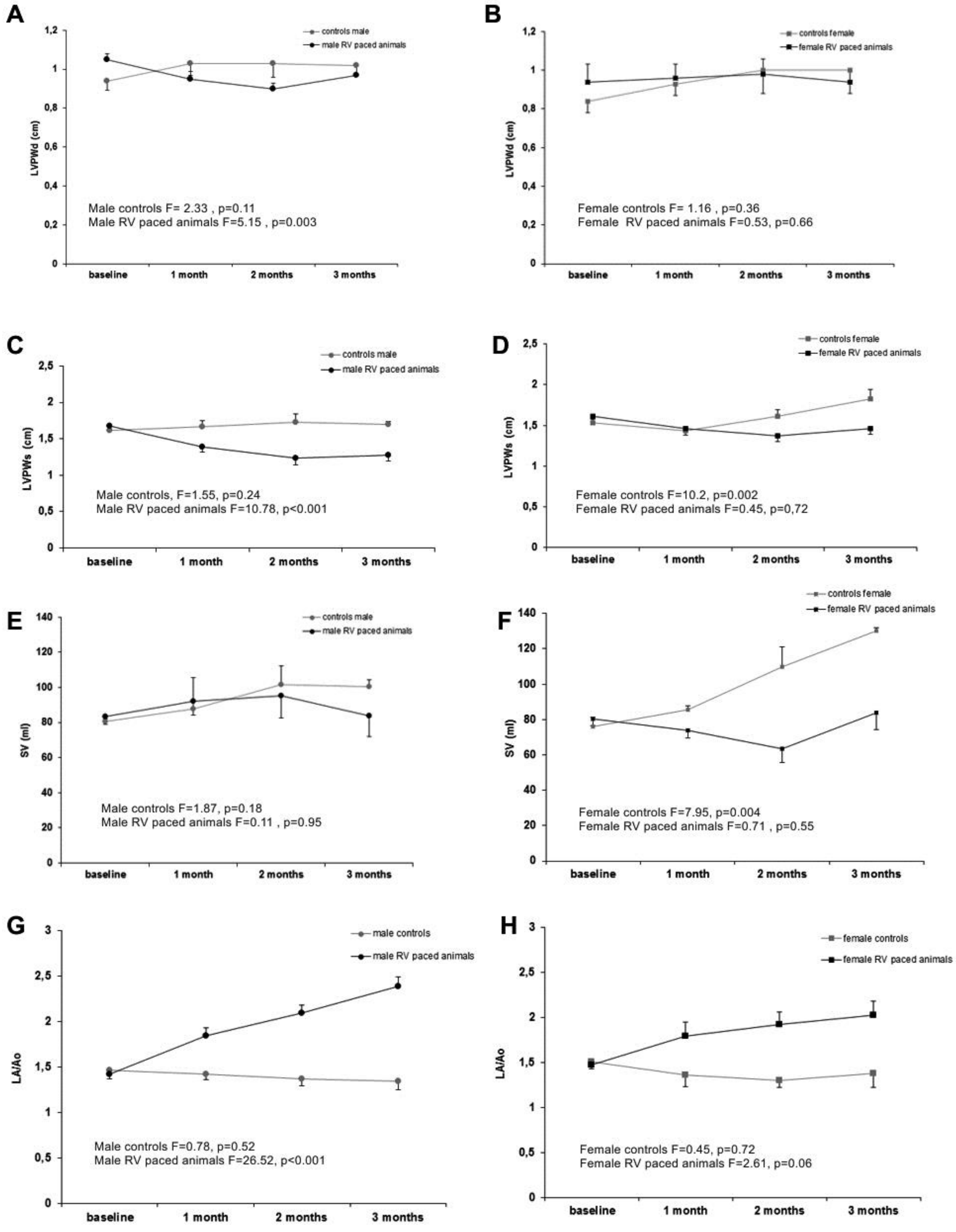
A
controls male
male RV paced animals
LVPWd (cm)
Male controls F= 2.33 , p=0.11
Male RV paced animals F=5.15 , p=0.003
baseline
1 month
2 months
3 months
B
controls female
female RV paced animals
LVPWd (cm)
Female controls F= 1.16 , p=0.36
Female RV paced animals F=0.53, p=0.66
C
LVPWs (cm)
Male controls, F=1.55, p=0.24
Male RV paced animals F=10.78, p<0.001
D
LVPWs (cm)
Female controls F=10.2, p=0.002
Female RV paced animals F=0.45, p=0,72
E
SV (ml)
Male controls F=1.87, p=0.18
Male RV paced animals F=0.11 , p=0.95
F
SV (ml)
Female controls F=7.95, p=0.004
Female RV paced animals F=0.71 , p=0.55
G
male controls
male RV paced animals
LA/Ao
Male controls F=0.78, p=0.52
Male RV paced animals F=26.52, p<0.001
H
female controls
female RV paced animals
LA/Ao
Female controls F=0.45, p=0.72
Female RV paced animals F=2.61, p=0.06

Figure S1.

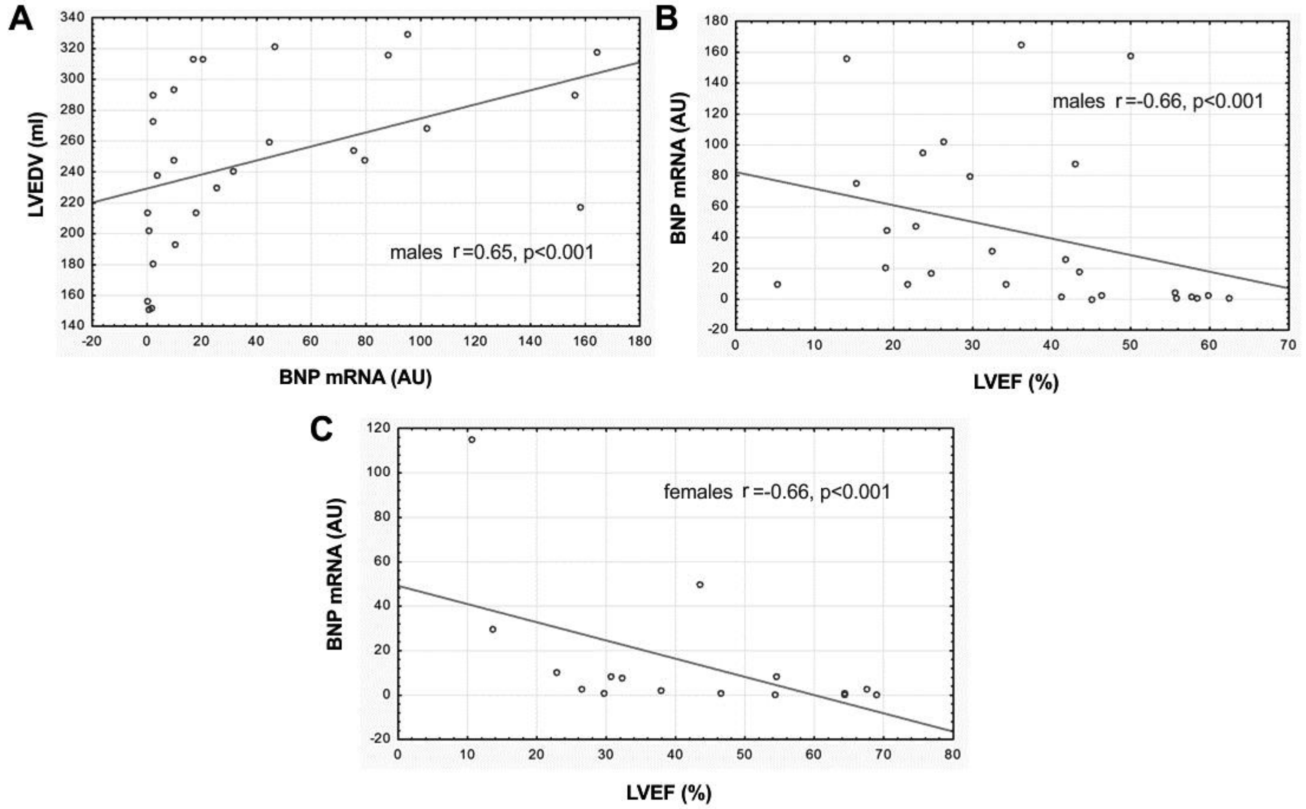

A
LVEDV (ml)
BNP mRNA (AU)
males r=0.65, p<0.001
B
BNP mRNA (AU)
LVEF (%)
males r=-0.66, p<0.001
C
BNP mRNA (AU)
LVEF (%)
females r=-0.66, p<0.001

Figure S2.

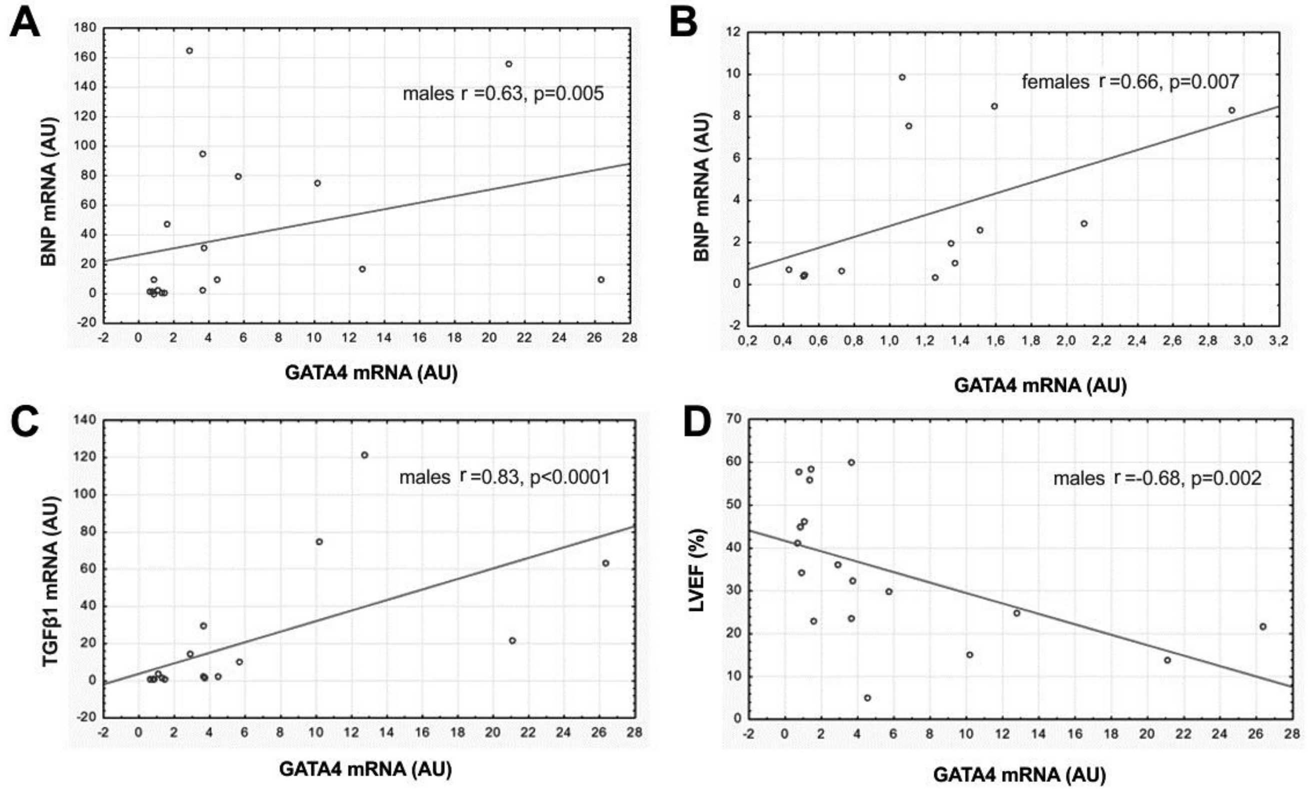
A
males r =0.63, p=0.005
BNP mRNA (AU)
GATA4 mRNA (AU)
B
females r =0.66, p=0.007
BNP mRNA (AU)
GATA4 mRNA (AU)
C
males r =0.83, p<0.0001
TGFβ1 mRNA (AU)
GATA4 mRNA (AU)
D
males r =-0.68, p=0.002
LVEF (%)
GATA4 mRNA (AU)

Figure S3.

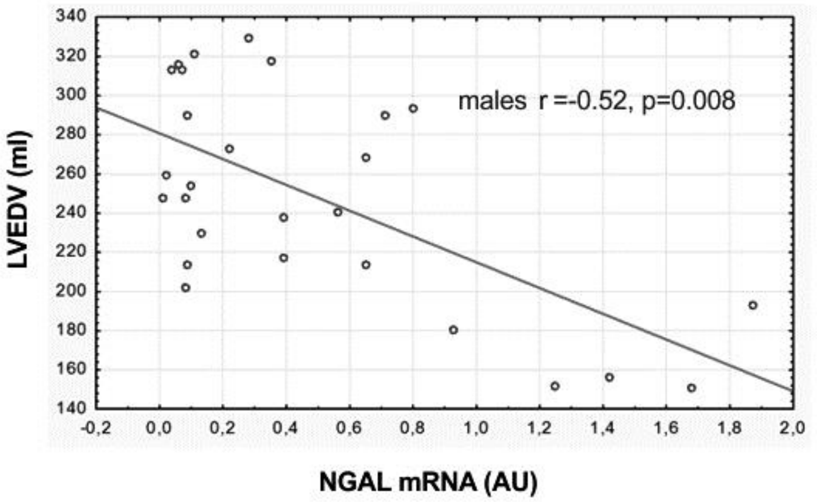

males r =-0.52, p=0.008
LVEDV (ml)
NGAL mRNA (AU)
340
320
300
280
260
240
220
200
180
160
140
-0,2
0,0
0,2
0,4
0,6
0,8
1,0
1,2
1,4
1,6
1,8
2,0

Figure S4.

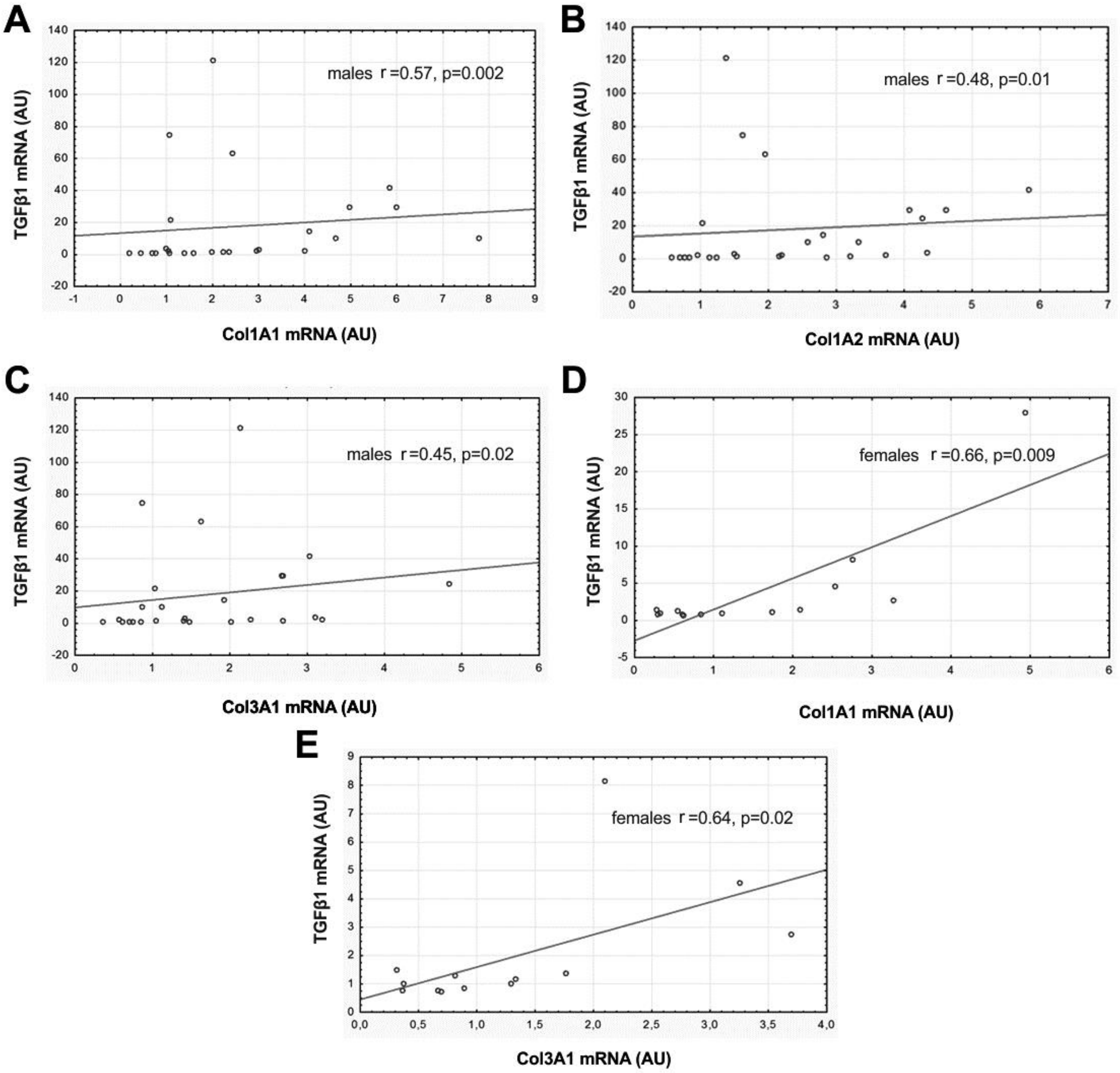
A
males r =0.57, p=0.002
TGFβ1 mRNA (AU)
Col1A1 mRNA (AU)
B
males r =0.48, p=0.01
TGFβ1 mRNA (AU)
Col1A2 mRNA (AU)
C
males r =0.45, p=0.02
TGFβ1 mRNA (AU)
Col3A1 mRNA (AU)
D
females r =0.66, p=0.009
TGFβ1 mRNA (AU)
Col1A1 mRNA (AU)
E
females r =0.64, p=0.02
TGFβ1 mRNA (AU)
Col3A1 mRNA (AU)

Figure S5.

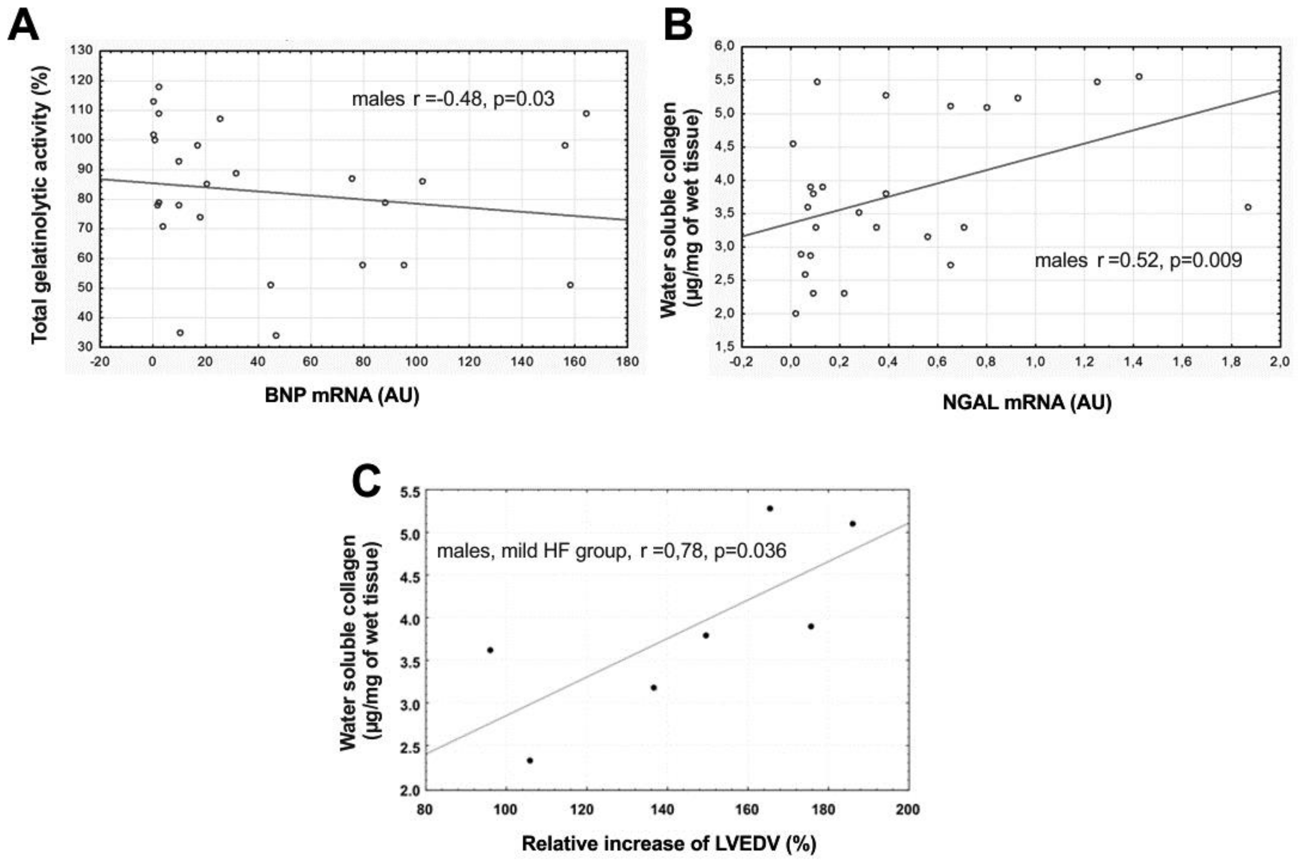
A
Total gelatinolytic activity (%)
males r =-0.48, p=0.03
BNP mRNA (AU)
B
Water soluble collagen
(µg/mg of wet tissue)
males r =0.52, p=0.009
NGAL mRNA (AU)
C
Water soluble collagen
(µg/mg of wet tissue)
males, mild HF group, r =0,78, p=0.036
Relative increase of LVEDV (%)

Figure S6.

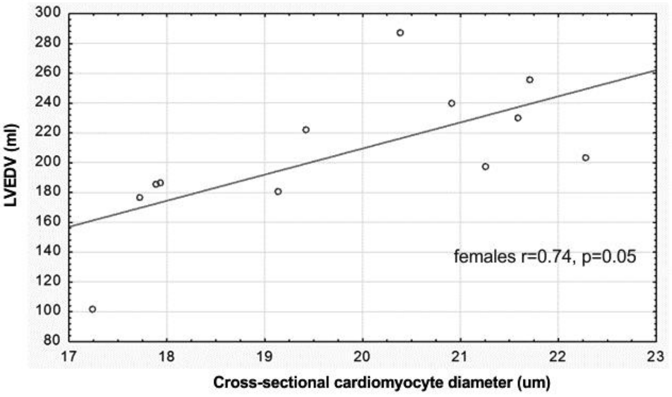

300
280
260
240
220
200
180
160
140
120
100
80
LVEDV (ml)
17
18
19
20
21
22
23
Cross-sectional cardiomyocyte diameter (um)
females r=0.74, p=0.05

Figure S7.

Table S1 Initial values of echocardiography parameters in sham-operated male pigs (controls) and right ventricle paced pigs with induced heart failure.

	female	male	
Initial LVEF, %	61.3±9.4	57.3±6.5	ns
Initial LVEDV, ml	129.1±29.5	148.8±20	ns
Initial La/Ao	1.46±0.2	1.43±0.16	ns
Initial Em/Am	1.52±0.22	2.20±0.75	p=0.001
Initial LVPWd, cm	0.92±0.14	0.99±0.16	ns
Initial LVPWs, cm	1.55±0.21	1.63±0.22	ns
Initial RWTd	0.35±0.06	0.37±0.06	ns
Initial SV, ml	85.5±26.9	81.8±17.7	ns

LVEF – left ventricular ejection fraction; LVEDV – left ventricular end-diastolic volume; LA/Ao - left atrial/aorta ratio; Em/Am – early diastolic to late diastolic velocity ratio, LVPWd – end-diastolic thickness of left ventricle posterior wall; LVPWs – end-systolic thickness of left ventricle posterior wall; RWTd – relative wall thickness at end diastole (2xLVPWd/LVEDD, LVEDD – left ventrical end-diastolic diameter); SV – stroke volume. All echocardiography measures were performed at t=0. Data are presented as means ± SD.

Table S2 Echocardiography parameters reflecting the structure and functioning of left ventricle in sham-operated male pigs (controls) and right ventricle paced pigs with induced heart failure.

	LVPWs, cm	LVPWd, cm	SV, ml
controls, females	1.60±0.35	0.92±0.06	112.3±23.4
mild HF, females	1.30±0.14	0.95±0.15	77.08±19.9
moderate HF, females	1.62±0.12	0.95±0.15	92.2±35.9
severe HF, females	1.26±0.19	1.06±0.08	46.5±18.7
controls, males	1.65±0.28	1.08±0.19	115.5±35.2
mild HF, males	1.39±0.26	0.93±0.13	107.07±32.7$^{\&}$
moderate HF, males	1.35±0.29	0.97±0.11	111.5±35.6
severe HF, males	1.13±0.14*	0.96±0.17	70.6±33*

HF – heart failure; LVPWs – end-systolic thickness of left ventricle posterior wall; LVPWd – end-diastolic thickness of left ventricle posterior wall; SV – stroke volume. All echocardiography measures were performed directly before an euthanasia. Data are presented as means ± SD.

*, p<0.05 vs. control group;

$^{\&}$, p<0.05 vs. corresponding female group.

Table S3 Sex related differences in selected echocardiography parameters assessed in left ventricular myocardium in male pigs with and without tachycardia-induced cardiomyopathy (the results of the two-way ANOVA).

Variables (units)	Sex		HF groups		Interactions (sex and HF groups)	
	F	p	F	p	F	p
SV, ml	2.08	0.16	1.57	0.21	0.48	0.70
LVPWs (cm)	0.55	0.46	3.95	0.016	0.98	0.41
LVPWd (cm)	0.02	0.89	0.75	0.53	1.53	0.22

SV – stroke volume; LVPWs – end-systolic thickness of left ventricle posterior wall; LVPWd – end-diastolic thickness of left ventricle posterior wall.

Table S4 Relationships between selected echocardiographic parameters and HF insufficiency in male and female pigs with and without tachycardia-induced cardiomyopathy (the results of Spearman's rank correlatory coefficients).

Variables (units)	male		female		sex controls
	r	p	r	p	p
SV (ml)	-0.39	0.04	-0.35	0.17	
LVPWd (cm)	-0.21	0.28	-0.19	0.47	
LVPWs (cm)	-0.49	0.009	-0.19	0.47	

SV – stroke volume; LVPWd – end-diastolic thickness of left ventricle posterior wall; LVPWs – end-systolic thickness of left ventricle posterior wall.
